# “Anti-obesity medications” or “medications to treat obesity” instead of “weight loss drugs” – why language matters – an official statement of the Brazilian Association for the Study of Obesity and Metabolic Syndrome (ABESO) and the Brazilian Society of Endocrinology and Metabolism (SBEM)

**DOI:** 10.20945/2359-4292-2023-0174

**Published:** 2023-08-16

**Authors:** Bruno Halpern, Marcio C. Mancini, Simone van de Sande-Lee, Paulo Augusto Carvalho Miranda

**Affiliations:** 1 Associação Brasileira para o Estudo da Obesidade e Síndrome Metabólica São Paulo SP Brasil Associação Brasileira para o Estudo da Obesidade e Síndrome Metabólica, São Paulo, SP, Brasil; 2 Sociedade Brasileira de Endocrinologia e Metabologia Departamento de Obesidade São Paulo SP Brasil Departamento de Obesidade, Sociedade Brasileira de Endocrinologia e Metabologia, São Paulo, SP, Brasil; 3 Hospital 9 de Julho Centro de Obesidade São Paulo SP Brasil Centro de Obesidade, Hospital 9 de Julho, São Paulo, SP, Brasil; 4 Faculdade de Medicina da Universidade de São Paulo Departamento de Endocrinologia e Metabolismo Grupo de Obesidade e Síndrome Metabólica São Paulo SP Brasil Grupo de Obesidade e Síndrome Metabólica, Departamento de Endocrinologia e Metabolismo, Faculdade de Medicina da Universidade de São Paulo, São Paulo, SP, Brasil; 5 Universidade Federal de Santa Catarina Departamento de Clínica Médica Florianópolis SC Brasil Departamento de Clínica Médica, Universidade Federal de Santa Catarina, Florianópolis, SC, Brasil; 6 Sociedade Brasileira de Endocrinologia e Metabologia São Paulo SP Brasil Sociedade Brasileira de Endocrinologia e Metabologia, São Paulo, SP, Brasil; 7 Santa Casa de Belo Horizonte Belo Horizonte MG Brasil Santa Casa de Belo Horizonte, Belo Horizonte, MG, Brasil

**Keywords:** Obesity, weight loss, treatment, drugs, language

## Abstract

Obesity is largely undertreated, in part because of the stigma surrounding the disease and its treatment. The use of the term “weight loss drugs” to refer to medications for the treatment of obesity may contribute to this stigma, leading to the idea that anyone who wants to lose weight could use them and that short-term use, only in the active weight loss phase would be enough. On the contrary, the use of terms such as “medications to treat obesity” or “anti-obesity medications” conveys the idea that the treatment is directed at the disease rather than the symptom. This joint statement by the Brazilian Association for the Study of Obesity and Metabolic Syndrome (ABESO) and the Brazilian Society of Endocrinology and Metabolism (SBEM) intends to alert the press, healthcare professionals and scientific community about the importance of the appropriate use of language, with the aim of improving obesity care.

## INTRODUCTION

Obesity is a common chronic disease associated with several comorbidities, disability and mortality as well as low quality of life (1,2); however, it is still widely underdiagnosed and undertreated (3). Obesity stigma is highly prevalent, as well as the stigma against its treatment, whatever medical or surgical (4-6). A few years ago, an editorial in *Expert Opinion on Drug Safety* discussed some of the reasons why obesity pharmacotherapy is stigmatized (5).

Part of the reasons can be attributed to the widespread idea that, rather than treating obesity itself, those drugs are “weight loss medications”; as such, seen as drugs that should be used on a short-term basis, only during the acute weight loss period (5). Moreover, when we refer to these medications as “weight loss drugs”, we contribute to the concept that their use has an aesthetic goal and can be consumed by anyone who desires to lose weight. In this short article, we would like to emphasize why the scientific community, as well as the media, should definitely stop using the term “weight loss medications” and start using “medications to treat obesity”, “anti-obesity medications” or some similar terms that emphasize that the treatment is aimed at a disease rather than a symptom.

### The obesity pharmacotherapy stigma

Despite the known health and economic burden of obesity, pharmacological treatment is widely underused (3,7-10). In 2015, in the US, only one in 50 patients with obesity received a prescription (9). A more recent analysis suggested a slight increase in prescriptions, reaching 3% of adults with obesity in 2019 (10). In 2016, the number of prescriptions dispensed for diabetes (excluding insulin) was 15 times greater than the number of prescriptions for the treatment of obesity (8). Even when considering programs focusing on weight loss for individuals with obesity, almost all attention is given to lifestyle changes. In a weight management program called MOVE!, centered in US veterans with overweight or obesity, only 1.1% received an obesity medication prescription, being orlistat the most prescribed drug, reaching 70% of the total prescribed medications (7). Moreover, a recent market study suggested that 50% of patients with obesity never received an anti-obesity medication prescription, and, when prescribed, the treatment maintenance after 12 months was as low as 2% (11). Moreover, even when anti-obesity medication is taken, persistence is low (12).

In the ACTION-IO study, that revealed thoughts and perceptions of both healthcare professionals (HCPs) and patients living with obesity (PwO), only 40% of PwO considered medications an efficacious option compared to 30% of HCPs; moreover, medications were discussed in only 18% of visits (13). Nevertheless, lifestyle modifications alone were perceived as effective by almost 80% of both PwO and HCPs, despite the fact that evidence points to a limited effect of these interventions in isolation (14,15). As an example, a highly cited and well conducted meta-analysis showed that the mean mid- to long-term weight loss achieved with comprehensive lifestyle modification programs is around 3 kg (16). Other studies show that only 10% of patients are able to lose and sustain a 10% weight loss after one to two years in intensive programs, but their combination with medications can significantly improve the results, as several well conducted randomized clinical trials (RCTs) have concluded (17-20). It is noteworthy, however, that the ACTION-IO responses from HCPs clearly point out that high grade evidence from RCTs are not being used to drive HCPs decisions in obesity (13).

### Some reasons for the limited use of obesity pharmacotherapy and its stigmatization

In the aforementioned editorial in 2015, Halpern and Halpern discussed several reasons why there is stigma around antiobesity drugs by doctors, patients, public health agents, stakeholders and even regulatory agencies (5). These were: 1) the already mentioned idea that obesity is not a disease, but mostly a “choice”; 2) weight gain occurs after treatment is interrupted; 3) weight loss is lower than anticipated by patients and doctors; 4) drugs are commonly used for aesthetic reasons; 5) there is a perception that they are associated with many serious side effects and risks (and, indeed, several drugs have been withdrawn from the market in the last decades for safety reasons); 6) as a common disease, obesity is generally treated in primary care, where the training of HCPs regarding its treatment is often deficient.

Cost is also an important reason for the low use of medications in general (21), and, in obesity, this can be a major challenge for long-term adherence (12). This is particularly true in Brazil, where almost 100% of antiobesity medications are paid out-of-pocket, since there are no free antiobesity medications offered by the public health system and health insurances generally do not cover ambulatory medications (22); indeed, this scenario of low medication coverage for obesity is also the rule in several other countries (23). Discussions about availability of some of these medications in the public service have led to inaction, since the stigma is prevalent. One should consider as well that costs of incorporation could be very high, due to the high prevalence of obesity in the adult population (22). However, since obesity is associated with higher morbidity and mortality, it can be argued that treating it could reduce direct and indirect costs (24,25). Moreover, even with the availability of some medications, lack of training on obesity in medical schools could result in their incorrect use (5,26). A recent survey in the US found that less than 10% of doctors make use of obesity guidelines to substantiate their treatment decisions (26). There is no doubt however, that the main reason for antiobesity medication rejection is the stigma of obesity itself (4,5,27).

### Weight stigma and the importance of language

Stigma in healthcare is very common in several scenarios and populations, as in individuals with infectious diseases, disabilities, mental illness, among others (4,28,29). Weight stigma, defined as negative attitudes and actions towards people with overweight or obesity, impairs health and well-being and is perceived in settings as in the workplace, in school, at home and even in healthcare settings (4,29). In an online Brazilian survey sponsored by both ABESO and SBEM societies, it was noted that in individuals with obesity 72% suffered embarrassment at home by their relatives, 60% in healthcare facilities and 55% at work (30). This number is higher in individuals with higher BMIs, and, in those with BMI over 40 kg/m^2^, 98% had experienced embarrassment at some point, and 25% reported daily embarrassment (30).

Among the several consequences of such negative attitudes is weight stigma internalization (29). Internalized weight stigma (IWS) refers to negative attitudes and thoughts about oneself (self-stigma), in which people with obesity believe and act as if these stereotypes are correct. Individuals with higher IWS are at risk of binge- and emotional eating, further weight gain and several health complications (4,29,31).

As such, health professionals should recognize they are also subject to weight bias, and that the way they communicate with patients could have a profound effect on health-related outcomes (4). Furthermore, weight-related stigma, as opposed to other stigmas, does not seem to be decreasing, and as obesity is commonly seen as an individual’s fault, this could lead to inaction by governments and other stakeholders in both prevention and treatment strategies (32). Indeed, withdrawal of some medications by regulatory agencies may have been, at least partially, influenced by weight stigma, and the fact that many medications have been withdrawn in the past directly impacts investment on new drugs (5,27).

The importance of language has been highlighted in the current effort to reduce weight-related stigma, and has been an issue in several obesity and diabetes journals (33-36), in guidelines (4,34-39), as well as in intersectoral meetings, documents and even a whole book in Brazil (40,41).

Several issues emerge on how to correctly communicate with patients – avoiding the use of judgmental words, for example (38). One of the most critical points is the promotion of the use of “people first” language (33,35,37,42). The understanding is that an individual should not be defined by their disease (as by the use of the terms “obese” or “diabetic”), but rather lives with this disease (“individual with obesity” or “with diabetes”) (33,42).

One particular aspect of chronic diseases such as obesity is that although they have no cure, they can be controlled (43-46). As such, an individual who had a high body mass index (BMI) and lost a considerable amount of weight, despite not fitting into the classification of obesity by BMI, should still have obesity (albeit controlled) as one of his or her diagnoses (45). Recently, ABESO and SBEM issued a proposal of a new obesity classification based on weight trajectory that highlights those points and which, in the opinion of both societies, helps to reduce stigma by highlighting that BMI “normalization” is not the goal of an obesity treatment, and that weight targets should be individualized (45,47).

Put together, we believe that the common use of the term “weight loss medications” by media and the general public, as well as by doctors and the scientific community, contributes to stigma, and, certainly, that “language matters” (33,34,40,42). As such, we propose that we should make an effort to abandon the use of “weight loss medications” in scientific publications, but most importantly, in the media (as its use is more widespread).

### “Medications to treat obesity” or “antiobesity medications” is extremely different from “weight loss medications”

In a simple Google search by June 2023, the term “weight loss drugs” leads to 2,200,000 results and “weight loss medications”, to an extra 630,000 results. On the other hand, a search for “antiobesity medications”, “antiobesity drugs”, or “drugs (or medications) to treat obesity”, leads to only 428,000 results, or 14% of the first search. “Obesity medications (or drugs)” leads to 170,000 extra results, but the term can be misleading. Of course, differences in interpretation exist between terms in different languages, but this search is a good example of the most common terms used on a public database. In academic databases, fortunately, the scenario changes a little. PubMed uses, in its Medical Subject Headings (Mesh) Database, the term “anti-obesity agents”, in which more than 19,000 results appears, and “weight loss drugs/agents/medications” in PubMed leads to much fewer results (less than 500). As such, it can be concluded that the academic milieu is more aware of this difference (although weight-loss drugs is a term generally heard in conferences and medical communications), but there is a gap between the scientific production of knowledge in this area and how it is translated to the general public, especially in the media. As such, it is important that the academic community be aware of this difference and increases its efforts to improve language, bridge this gap and reduce stigma. But why does this matter and is it not simply a semantic point?

First of all, weight loss is just a small part of the treatment of obesity itself (38,45,48). Generally, after a short period of weight loss, the weight reaches a plateau, and if the weight loss achieved is considered adequate, the treatment of obesity continues in a weight maintenance phase (48). The withdrawal of medications in this period – what is very common, by the patient himself or by doctors’ recommendation – leads to weight regain, as we should expect with any chronic disease (48-50). The fact that diabetes or hypertension medication withdrawal may lead to impairment of glycemic and blood pressure control does not surprise anyone. In spite of this, with obesity there is a common misconception that weight regain is a treatment failure, rather than an expected recurrence of an untreated chronic disease (5). If we use the term “weight loss medications”, weight regain after withdrawal is a fair argument against their use. However, the understanding by HCPs and PwO that medications are useful for both weight reduction and maintenance may help a lot in long term adherence.

The main goal of treating obesity is not to “normalize BMI”, but rather to improve health and quality of life, which can be achieved by a weight loss of 5%-15% (45,48,51,52), and this concept is highlighted in the recent SBEM and ABESO proposal of a new obesity classification (45). When BMI “normalization” is the sole goal of treatment, there is a high probability of patient frustration once the weight reaches a plateau, leading to the idea that the medication does not work anymore and should be stopped (5,45,53). In fact, at the weight plateau, medication has achieved its maximal effect on weight reduction and weight maintenance during treatment is a sign that it is still working (50).

Furthermore, the term “weight loss medications” does not discern who should be treated and treating obesity could be misconceived with treating the “social desire of weight loss” that is widespread in society (5). This contributes to the view that the drugs are mostly used for aesthetic reasons (and by many people who do not need them) and not for treating a disease associated with health and psychological burdens.

Additionally, we should not forget that treating obesity is more than just managing weight, as endorsed by the Canadian Clinical Practice Guideline, which had multiple positive reviews (38). Focus on mental health, reduction of internalized stigma, treatment of comorbidities, promotion of physical exercise (that improves health independently of the weight loss itself), setting long terms goals and targets, among others, are essential parts of the treatment (38). As such, drugs are just one of several strategies to tackle a chronic disease, and they can also help to reduce binge episodes or loss of eating control in addition to control hunger and increase satiety, and also to improve metabolic markers and comorbidities, independently of weight loss (54-58). Indeed, there is good evidence that at least some of these medications are able to reduce cardiovascular risk markers and improve obesity-related diseases, although there is a wide variability of effects depending on the mechanisms of action of each drug (59-65). Unfortunately, we do not have direct evidence that these drugs reduce cardiovascular or other hard outcomes in PwO, but this may change in the future, as newer trials aim to answer these questions (66,67).

Finally, we should differentiate regulatory agency-approved medications from over-the-counter drugs and supplements that are often sold as “weight loss agents” and are responsible for an unacceptably high rate of emergency visits (68,69). Using “anti-obesity pharmacotherapy” may help to undo this misconception, by reminding us that a drug to be used continuously to treat a chronic disease should be submitted to a high level of safety scrutiny as when approved by regulatory agencies (27).

One potential downside of emphasizing “obesity medications” is its understanding in the context of overweight individuals who can nevertheless also benefit from treatment. Guidelines and label indications vary by medication and country, but individuals over 25 or 27 kg/m² with obesity-related diseases are candidates for anti-obesity medications, despite not be affected by obesity by BMI criteria (38,48,51,52,70). However, although obesity is still diagnosed by BMI, several guidelines point out that BMI has many limitations on an individual basis, and obesity should be defined by its health impact (38,70-72). Indeed, obesity has been defined by the World Health Organization as an “excess fat accumulation that impairs health” (73). As such, using this concept, an individual who is overweight and has comorbidities may be considered as having clinical obesity, and the indication of long-term medication use, in this case, is similar to that for an individual with a higher BMI. A commission has recently been established by the American Association of Clinical Endocrinologists and the American College of Endocrinology to define obesity and establish its diagnosis independently of strict BMI thresholds (71).

Regarding other chronic diseases, such as diabetes, hypertension or hyperlipidemia, different ways of naming medications also arise, but these are generally less stigmatized conditions, in which the nomenclature may be less important for treatment perception. Nevertheless, in hypertension, “anti-hypertensive medications” is more used than “blood pressure reduction medications”, and “hypotensive medications” is seldom used; in diabetes “anti-diabetic” is still more common than “glucose-lowering” or “anti-hyperglycemic”. One exception is in hypercholesterolemia, in which “lipid-lowering drugs” is a common and widely used term, although in the Mesh database, the correct term would be “anticholesterolemic agents”. However, the stigmatization of hyperlipidemia is almost non-existent (28,74). Interestingly, non-adherence to anticholesterolemic agents is common, as well as their interruption after blood cholesterol goes down (74), and it is possible that pointing out the long-term importance not only of cholesterol reduction itself, but also of prevention of cardiovascular disease, may contribute to greater adherence to treatment (75,76). Thus, although this discussion regarding obesity seems more urgent to improve perceptions of treatment and reduce stigma, it does not imply that language is not interfering in the treatment of other diseases as well. In each case, critical reflection is needed on the reasons for choosing specific terms over others.

In Table 1, we summed up the main arguments for the correct use of language in this context.

**Table 1:** Reasons why language matters in obesity treatment

Term	Weight loss drugs	Antiobesity medications
Language adequacy	Inappropriate	Appropriate
Utilization scenario	Lay media	Academia, scientific publications
Targeting of the treatment	Symptom	Disease
Effect on stigma and medical care	Generate stigma	Obesity care improvement
Usage time	Short term use	Long term treatment
Insight of the term	Aesthetic goal	Scientific treatment of a chronic disease
Purpose of use	Social desire of weight loss	Treatment of a disease
Safety scrutiny	Low	High
Associated image	Over-the-counter drugs and supplements	Regulatory agency-approved medication

In conclusion, in obesity, words matter and the way we disseminate messages can either help individuals seeking support or perpetuate stigma. In addition, the way we name things leads to huge differences in how they are perceived and can change our perspective. We believe that a “call to action” to disseminate the importance of the avoidance of the term “weight loss drugs” in media and scientific publications, and the widespread use of “anti-obesity medications”, or “medications to treat obesity” is essential to help to reduce the stigma and improve adherence and persistence in obesity treatment.
